# Abnormal composition of gut microbiota contributes to delirium‐like behaviors after abdominal surgery in mice

**DOI:** 10.1111/cns.13103

**Published:** 2019-01-24

**Authors:** Jie Zhang, Jiang‐Jiang Bi, Guo‐Jun Guo, Ling Yang, Bin Zhu, Gao‐Feng Zhan, Shan Li, Nian‐Nian Huang, Kenji Hashimoto, Chun Yang, Ai‐Lin Luo

**Affiliations:** ^1^ Department of Anesthesiology, Tongji Hospital, Tongji Medical College Huazhong University of Science and Technology Wuhan China; ^2^ Department of Hand Surgery, Union Hospital, Tongji Medical College Huazhong University of Science and Technology Wuhan China; ^3^ Department of Cardiology and Critical Care Medicine The Third Affiliated Hospital of Soochow University Changzhou China; ^4^ Division of Clinical Neuroscience Chiba University Center for Forensic Mental Health Chiba Japan

**Keywords:** abdominal surgery, gut microbiota, gut‐brain axis, microbiota transplant, postoperative delirium

## Abstract

**Aims:**

Anesthesia and surgery can cause delirium‐like symptoms postoperatively. Increasing evidence suggests that gut microbiota is a physiological regulator of the brain. Herein, we investigated whether gut microbiota plays a role in postoperative delirium (POD).

**Methods:**

Mice were separated into non‐POD and POD phenotypes after abdominal surgery by applying hierarchical clustering analysis to behavioral tests. Fecal samples were collected, and 16S ribosomal RNA gene sequencing was performed to detect differences in gut microbiota composition among sham, non‐POD, and POD mice. Fecal bacteria from non‐POD and POD mice were transplanted into antibiotics‐induced pseudo‐germ‐free mice to investigate the effects on behaviors.

**Results:**

α‐diversity and β‐diversity indicated differences in gut microbiota composition between the non‐POD and POD mice. At the phylum level, the non‐POD mice had significantly higher levels of Tenericutes, which were not detected in the POD mice. At the class level, levels of Gammaproteobacteria were higher in the POD mice, whereas the non‐POD mice had significantly higher levels of Mollicutes, which were not detected in the POD mice. A total of 20 gut bacteria differed significantly between the POD and non‐POD mice. Interestingly, the pseudo‐germ‐free mice showed abnormal behaviors prior to transplant. The pseudo‐germ‐free mice that received fecal bacteria transplants from non‐POD mice but not from POD mice showed improvements in behaviors.

**Conclusions:**

Abnormal gut microbiota composition after abdominal surgery may contribute to the development of POD. A therapeutic strategy that targets gut microbiota could provide a novel alterative for POD treatment.

## 1 INTRODUCTION

Delirium is an acute abnormal change in cognitive function that is clinically characterized by alterations in consciousness with time fluctuations and unfocused attention.^1^ Postoperative delirium (POD) refers to delirium that occurs in patients who have undergone surgery.^2^, ^3^ It has been reported that POD mainly occurs at 24‐72 hours after surgery; importantly, it can increase the length of hospital stay and medical expenses, and it is associated with the increased incidence of short‐ and long‐term complications.^4^, ^5^ However, the recognition rate for POD is relatively low. It has been reported that approximately 35% of patients with delirium in an intensive care unit do not receive adequate medical attention or appropriate treatment.^6^ The early identification and diagnosis of POD are important.

The pathogenesis of POD is still under investigation. It has been proposed that therapeutic strategies targeted at reducing overactive inflammatory responses and oxidative stress or at improving dysfunctional cholinergic neurons can prevent and treat POD (Table S1).^7^, ^8^ Furthermore, it is well established that the incidence of POD significantly varies with the type of surgery. The incidences are less in patients who undergo minor or day‐case surgery,^9^ whereas they are up to 50% in patients who undergo major abdominal surgery.^10^, ^11^ The reasons underlying the high incidence of POD after abdominal surgery are not yet known.

The gut provides a living environment for microbiota growth and development. The adult human gut contains 10^12^‐10^14^ microbes, which is greater than the number of microorganisms on human skin and 10 times the number of cells in the body.^12^ Importantly, the gut microbiota has approximately 100 times the number of genes as the whole of the rest of the human body.^13^ Therefore, the human gut microbial community might be thought of as a functional organ in the human body or second human genome.^14^ The gut‐brain axis, a complex bidirectional signaling system between the gut and brain, plays a crucial role in brain function.^14^, ^15^ Increasing evidence suggests that gut microbiota remotely regulates brain functions.^15^ Abnormalities in gut microbiota composition have been reported in patients with autism or depression, with observed improvements to these neuropsychiatric symptoms after modifying the dysfunctional gut microbiota by probiotic treatment.^16^, ^17^ Abdominal surgery, particularly gastrointestinal surgery, is detrimental to gut microbiota composition,^18^ and it has been reported that minor abdominal surgery in infants caused long‐term changes in the colonic microbiota composition.^19^ A recent systematic review of 10 studies that included a total of 677 patients suggested that the gut microbiota composition is significantly changed postoperatively.^20^

On the basis of these findings, we speculated that abnormalities in the gut microbiota composition after abdominal surgery contribute to the onset of POD. The aim of this study was to investigate whether the gut microbiota plays a role in the underlying mechanisms of POD. If so, this would support the theoretical possibility that detecting the gut microbiota composition is helpful for preventing and treating POD.

## 2 MATERIALS AND METHODS

### 2.1 Animals

A total of 71 C57BL/6J male mice, 8 weeks old and weighing approximately 25 g, were purchased from the Experimental Animal Center of Tongji Medical College (Wuhan, China) and provided with food and water ad libitum. All experimental protocols were performed in accordance with the National Institutes of Health guidelines and regulations. The experimental protocols were approved by the Committee for Animal Experiments of Tongji Medical College (Wuhan, China).

### 2.2 Anesthesia and abdominal surgery

The mice were randomly assigned to either the anesthesia + surgery group (A + S group) or the sham group. Each A + S group mouse received 1.4% isoflurane and 100% oxygen in a transparent acrylic chamber for 15 minutes, as described previously.^21^, ^22^ A mask was subsequently placed over the head of the mice to maintain the 1.4% isoflurane with 100% oxygen, monitoring the concentration of isoflurane with an infrared probe (OhmedaS/5 Compact; Datex‐Ohmeda, Louisville, KY). A simple laparotomy was performed. A longitudinal midline incision was made from the xiphoid to a point on the skin 0.5 cm proximal to the pubic symphysis, then of the abdominal muscles and finally the peritoneum. The wound was sutured layer by layer with 5‐0 vicryl thread. After the surgery, lidocaine cream was applied to the wound for the incision pain until all the experiments were completed. The mice were placed back in the chamber with a total time under anesthesia of up to 2 hours; they were then returned to their own cages with food and water ad libitum. The mice in the sham group were placed in a similar transparent acrylic chamber with 100% oxygen for 2 hours. The rectal temperature of all the mice was maintained at 37 ± 0.5°C using a heating blanket.

### 2.3 Behavioral tests

For 1 week before the start of the study, the mice were kept singly under controlled conditions (temperature, 22 ± 2°C; relative humidity, 55 ± 10%; with a 12‐hour light/dark cycle) to adjust to the new environment. Twenty‐four hours before the anesthesia and surgery, the basic behaviors of all the mice were measured using the open‐field test (OFT), elevated plus maze test (EPMT), and buried food test (BFT). These tests were repeated at 6 hour after the anesthesia and surgery. The mice were placed into the test environment for 1 hour before performing the tests and returned to their individual cages after completing the tests. All the apparatus was cleaned with 70% ethanol after the removal of each mouse, and gloves were changed for each mouse.

#### 2.3.1 Open‐field test

As described previously,^21^, ^22^ a mouse was gently placed at the center of an open‐field chamber (40 × 40 × 40 cm) constructed of pleiglas and left alone for 5 minutes. The movements of the mouse were monitored and analyzed using the EthoVision tracking system (Noldus Information Technology, Wageningen, the Netherlands). The following parameters were recorded: total distance moved (in meters), number of times the mouse crossed the center, time (in seconds) spent in the central area of the open field, and number of times the mouse crossed each zone.

#### 2.3.2 Elevated plus maze test

The maze included four arms, each 50 × 10 cm, arranged in a cross shape with a central region measuring 10 × 10 cm. The height from the floor was 50 cm. Two of the arms were completely open; the other two were enclosed at the sides and ended with 9‐cm‐high perspex walls. At the start of the test, the mouse was placed in the central region facing one of the open arms. The number of times the mouse entered each open or closed arm during a 5‐minute period was recorded. Entering an arm was defined as two paws crossing the dividing line between the central region and arm.^21^, ^23^

#### 2.3.3 Buried food test

The mouse was given a single piece of buttered bread 48 hours before the test. After finishing the EPMT, the mouse was placed in a clean cage with 3‐cm‐high padding in which a pellet of buttered bread was buried. The location was freely chosen and out of sight. The test started with the mouse at the center of the cage. Latency was measured as the time from then until the mouse found the pellet and took hold of it with its forepaws or teeth. If the mouse found the pellet within 5 minutes, it was permitted to eat it before being returned to its cage. If the mouse was unable to find the pellet within 5 minutes, it was returned to its cage and the latency was recorded as 300 seconds.^21^

### 2.4 16S rRNA analysis of fecal samples

**Figure 1:**
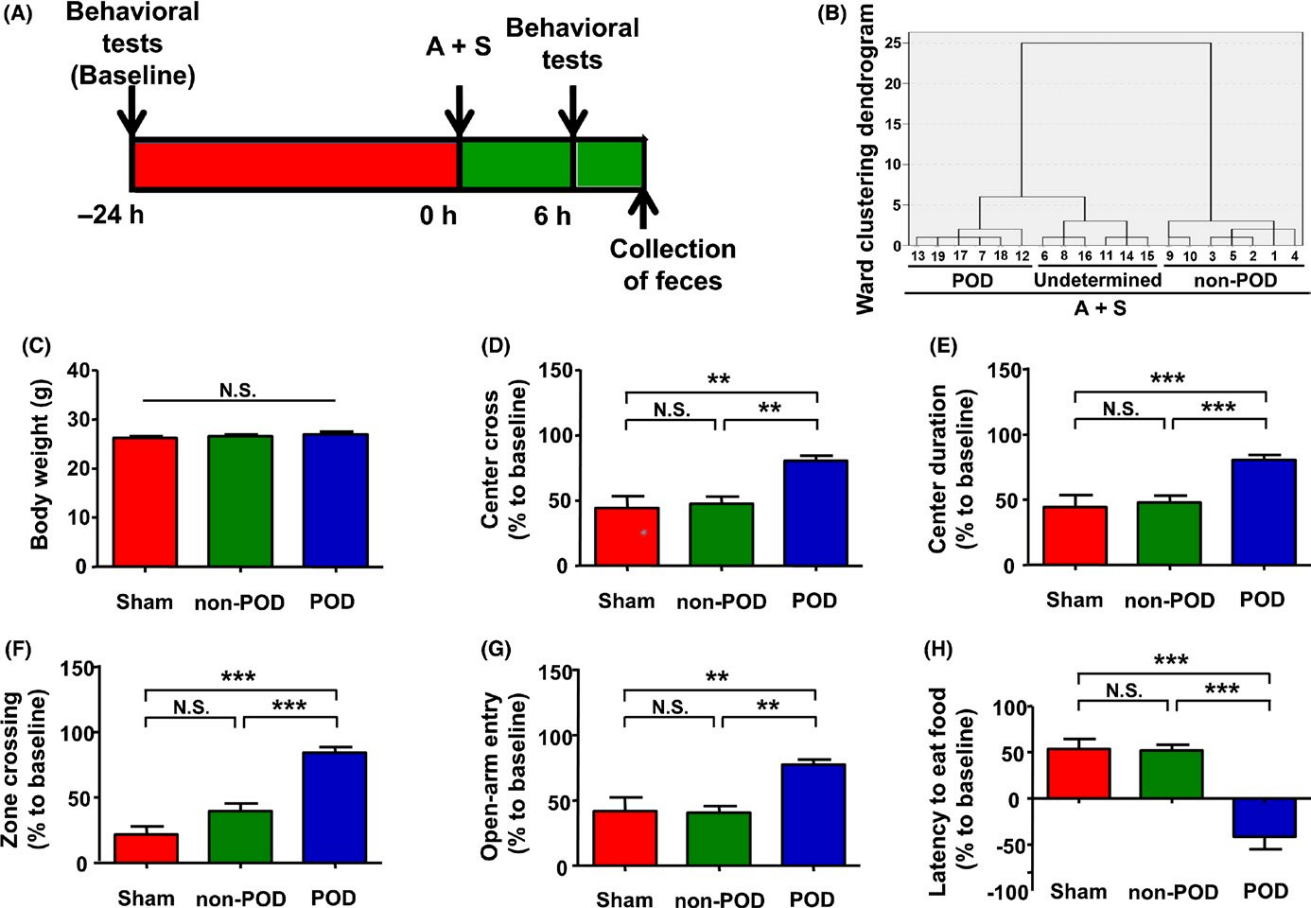
The schedule and behavioral tests. A, The study schedule. Behavioral tests, including the open‐field test, evaluated plus maze test, and buried food test, were performed 24 h before A + S, and 6 h after it. Fresh fecal samples were collected after all behavioral tests B, Dendrogram of the hierarchical clustering analysis. After A + S, 19 mice were classified into POD, non‐POD, and undetermined groups by hierarchical clustering analysis of the results of the behavioral tests. C, Body weight (one‐way ANOVA, *F*_2,16_ = 0.9403, *P* > 0.05). D, Center crossing (one‐way ANOVA, *F*_2,16_ = 8.27, *P* < 0.01). E, Time spent at the center (one‐way ANOVA, *F*_2,16_ = 14.49, *P* < 0.001). F, Zone crossing (one‐way ANOVA, *F*_2,16_ = 30.88, *P* < 0.001). G, Entries into the open arms (one‐way ANOVA, *F*_2,16_, *P* < 0.01). H, Latency to find the food pellet (one‐way ANOVA, *F*_2,16_ = 26.95, *P* < 0.001). A + S, anesthesia and abdominal surgery; ANOVA, analysis of variance; NS, not significant; POD, postoperative delirium. Data are shown as mean ± SEM (n = 6 or 7). **P < 0.01, ***P < 0.001

Fecal samples were collected after all behavioral tests (Figure 1A). These were placed in 1.5‐mL tubes, snap frozen on dry ice, and stored at −80°C prior to 16S rRNA analysis of the samples at Oe Biotech Co., Ltd., Shanghai, China. DNA extraction was performed using TIANamp Stool DNA Kits (Tiangen Biotechnology Company, Beijing, China). Genomic DNA was then amplified in 50‐μL triplicate reactions with primers specific to the V3‐V4 region of the bacterial 16S rRNA gene: 338F (5′‐ACTCCTACGGGAGGCAGC‐3′) and 806R (5′‐GG ACTACHVGGGTWTCTAAT‐3′). The reverse primer contained a sample barcode, and both primers were connected with an Illumina sequencing adaptor (Illumina company, San Digeo, CA, USA). The PCR products were purified, and the concentrations were adjusted for sequencing on an Illumina Miseq PE300 system. The original sequencing reads from the samples were sorted by the unique barcodes, and the barcodes, linkers, and PCR primer sequences were then removed. The resultant sequences were screened for quality, and 70 or more base pairs were selected for the bioinformatics analysis. All the sequences were classified using the NCBI BLAST and SILVA databases. Distance calculations, operational taxonomic unit clustering, rarefaction analysis, and estimator calculation (for α‐diversity and β‐diversity) were performed with the MOTHUR program.^24^

### 2.5 Pseudo‐germ‐free mouse model

Pseudo‐germ‐free mice were prepared as described in a previous study with slight modification.^25^ Broad‐spectrum antibiotics (ampicillin 1 g/L, neomycin sulfate 1 g/L, and metronidazole 1 g/L; Sigma‐Aldrich Co. Ltd, St. Louis, MO, USA) were dissolved in drinking water and given ad libitum to C57BL/6 mice for 14 consecutive days. The drinking solution was renewed every 2 days.

### 2.6 Fecal microbiota transplant

The mice were placed in a clean cage with sterilized filter paper. Immediately after defecation, fecal samples were collected in a sterilized centrifuge tube. The filter paper was replaced for each mouse. The samples were stored in a freezer at −80°C until analysis and transplant. The fecal microbiota was prepared by diluting 1‐g fecal sample obtained from either POD or non‐POD mice in 10 mL of sterile phosphate‐buffered saline. The fecal material was suspended, and 0.2 mL of the suspension was guided by gavage into each mouse recipient for 14 days.^25^

### 2.7 Statistical analysis

Data are presented as the mean ± standard error of the mean (SEM). Analysis was performed using PASW Statistics 20 (formerly SPSS Statistics; SPSS Inc, Chicago, IL). Comparisons among groups were performed using one‐way analysis of variance (ANOVA) followed by post hoc Tukey tests or Fisher's exact tests. Normal distribution data were analyzed using one‐way ANOVA, whereas non‐normal distribution data were analyzed using Fisher's exact test. After standardizing the data by *z* scores, hierarchical cluster analysis of the OFT, EPMT, and BFT results was performed using Ward's method, applying the squared Euclidean distance as the distance measure. This resulted in classification of the mice as POD‐susceptible, POD‐unsusceptible, and undetermined clusters. Principal coordinate analysis (PCoA) and principal components analysis (PCA) were performed to visualize the similarities and dissimilarities of the data for the three groups. *P* values <0.05 were considered statistically significant.

## 3 RESULTS

### 3.1 Effects of anesthesia and abdominal surgery on body weight and behavioral tests

First, hierarchical cluster analysis (Ward's method) was performed to classify the mice after the anesthesia and abdominal surgery into three clusters: POD‐susceptible, POD‐unsusceptible, and undetermined (Figure 1B). The results of behaviors were standardized by *z* scores. Six of 19 mice showed POD‐like phenotypes, whereas seven mice displayed non‐POD‐like phenotypes; the others were regarded as POD undetermined.

Next, we compared the body weight and results of behavioral tests, including OFT, EPMT, and BFT, among the sham, non‐POD, and POD groups (Figure 1C). There was no significant change in the body weight among the three groups. In OFT (Figure 1D‐F), the POD mice showed significant increases in center crossing, center duration, and zone crossing compared with those in the non‐POD group, whereas no change was found in OFT between the non‐POD and sham mice. The POD mice significantly increased open‐arm entry compared with the non‐POD and sham mice, but between the sham and non‐POD mice, there was no change (Figure 1G). Additionally, the POD mice, but not the non‐POD mice, significantly decreased the latency to eat food in BFT compared with the sham mice. Relative to the non‐POD mice, there was a decrease in latency to eat food in BFT in the POD mice (Figure 1H).

### 3.2 Comparison of gut microbiota composition among the sham, non‐POD, and POD groups

**Figure 2:**
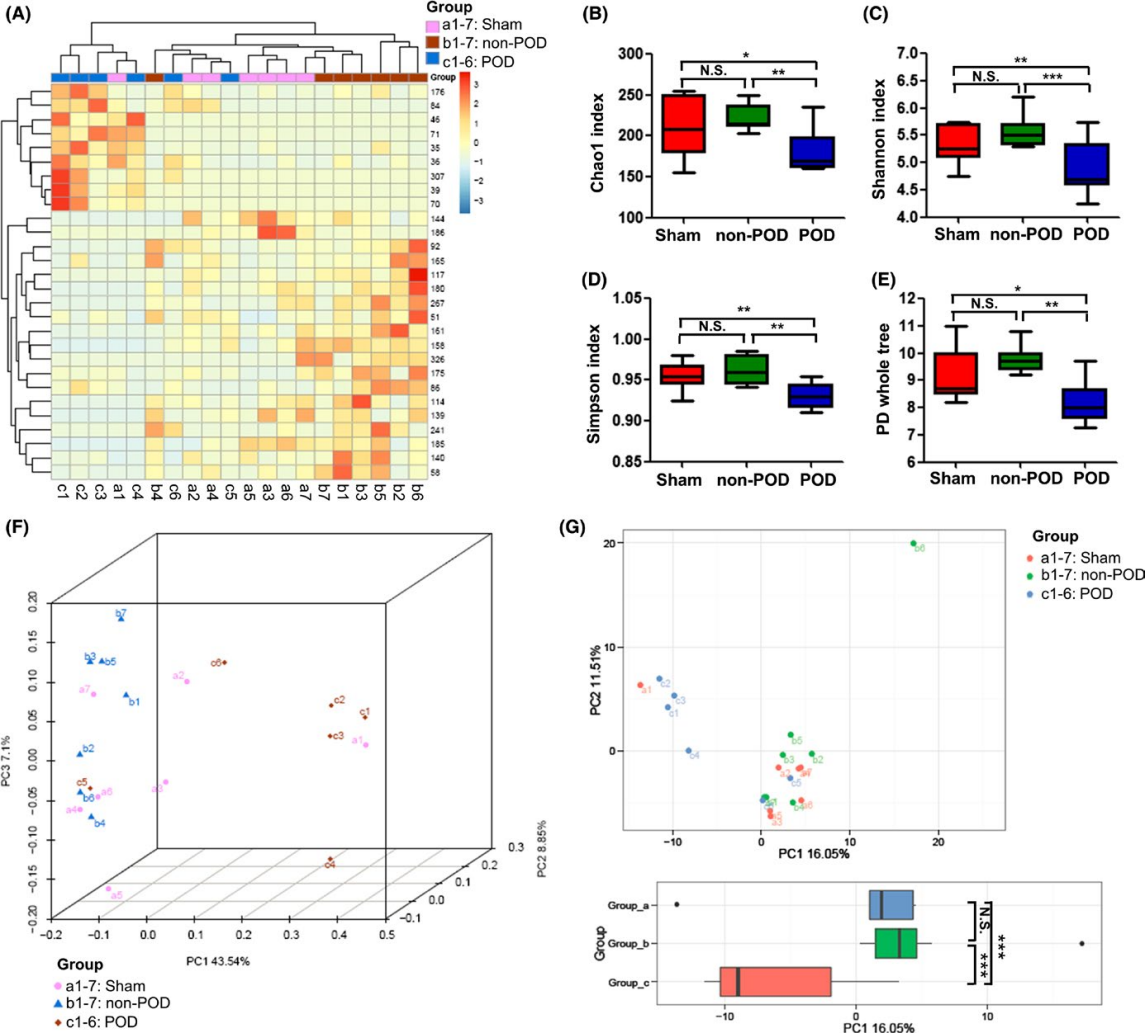
Differences in gut microbiota profiles between the groups. A, A heat map of the different levels of bacteria among the groups. *Y*‐axis: the number of operational taxonomic units; *X*‐axis: groups; a: sham group; b: non‐POD group; c: POD group. B, Chao 1 index (one‐way ANOVA, *F*_2,17_ = 10.607, *P* < 0.01). C, Shannon index (one‐way ANOVA, *F*_2,17_ = 16.767, *P* < 0.001). D, Simpson index (one‐way ANOVA, *F*_2,17_ = 6.621, *P* < 0.01). E, PD whole tree (one‐way ANOVA, *F*_2,17_ = 5.363, *P* = 0.016). F, PCoA analysis of the gut bacteria data (Bray‐Curtis dissimilarity). G, PCA analysis of the gut bacteria data (Bray‐Curtis dissimilarity, one‐way ANOVA, PC1: *F*_2,31_ = 14.909, *P* < 0.001). *P < 0.05, **P < 0.01 and ***P < 0.001

A heat map briefly described that the gut microbiota composition between the non‐POD and POD groups is completely different (Figure 2A). α‐Diversity refers to the diversity of bacteria or species within a community or habitat and is mainly concerned with the number of bacteria or species.^26^ We found that POD mice significantly decreased the Chao 1, Shannon, Simpson indices and PD whole tree compared with those in the non‐POD groups, whereas there was no change in α‐diversity indices between the sham and non‐POD mice (Figure 2B‐E).

In addition to α‐diversity, β‐diversity is also a parameter to evaluate the microbiota composition. β‐Diversity refers to the alternation rate of bacteria or species composition between different habitats along the environmental gradient; it is also known as between‐habitat diversity.^27^ The PCoA analysis plots of Bray‐Curtis dissimilarity among the three groups showed that the dots of the sham group (a1‐a7) were close to the dots of the non‐POD group. Most importantly, the dots of the POD group (a1‐a7) were far away from the dots of the non‐POD group (Figure 2F). Moreover, the POD mice significantly decreased the PCA (PC1) score compared with the non‐POD mice (Figure 2G).

Collectively, these findings suggest differential gut microbiota composition between the POD and non‐POD mice, and the number and types of bacteria in the gut of the POD and non‐POD mice were completely different.

### 3.3 Alterations in gut microbiota composition at the phylum level

**Figure 3:**
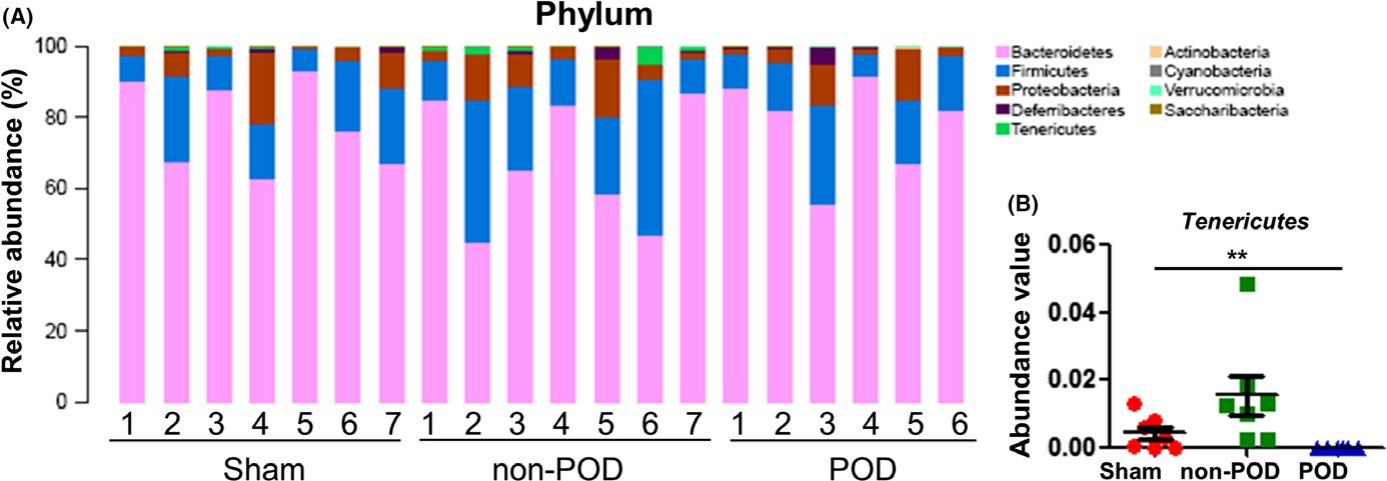
Changes in gut microbiota composition at the phylum level. A, Chart of the relative abundance of the differential levels of bacteria at the phylum level. B, Tenericutes level (Fisher's exact test, **P < 0.01)

The relative abundance chart (Figure 3A) of gut microbiota composition at the phylum level is shown. Fisher's exact test showed a significant change in the level of Tenericutes among the three groups (Figure 3B). Relative to the non‐POD mice, the POD mice showed a significant decrease in the level of Tenericutes.

### 3.4 Alterations in gut microbiota composition at the class level

**Figure 4:**
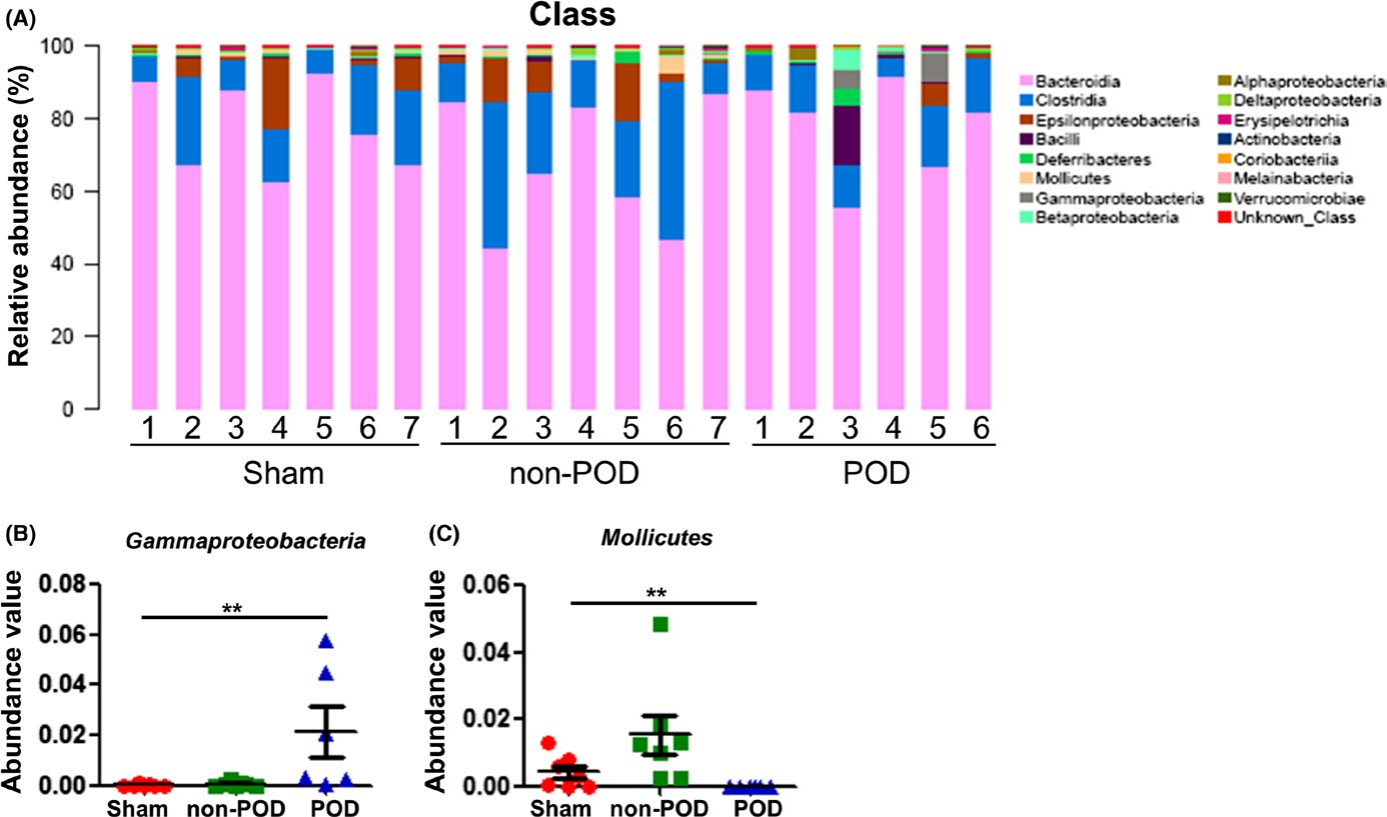
Changes in gut microbiota composition at the class level. A, Chart of the relative abundance of the differential levels of bacteria at the class level. B, Gammaproteobacteria level (Fisher's exact test, **P < 0.01). C, Mollicutes level (Fisher's exact test, **P < 0.01)

The relative abundance chart (Figure 4A) of gut microbiota composition at the class level is shown. A significant change in the levels of Gammaproteobacteria and Mollicutes was observed among the three groups. We found that the POD mice showed significantly increased levels of Gammaproteobacteria, but Mollicutes was not detected in the gut of the POD mice (Figure 4B,C).

### 3.5 Alterations in gut microbiota composition at the order level

**Figure 5:**
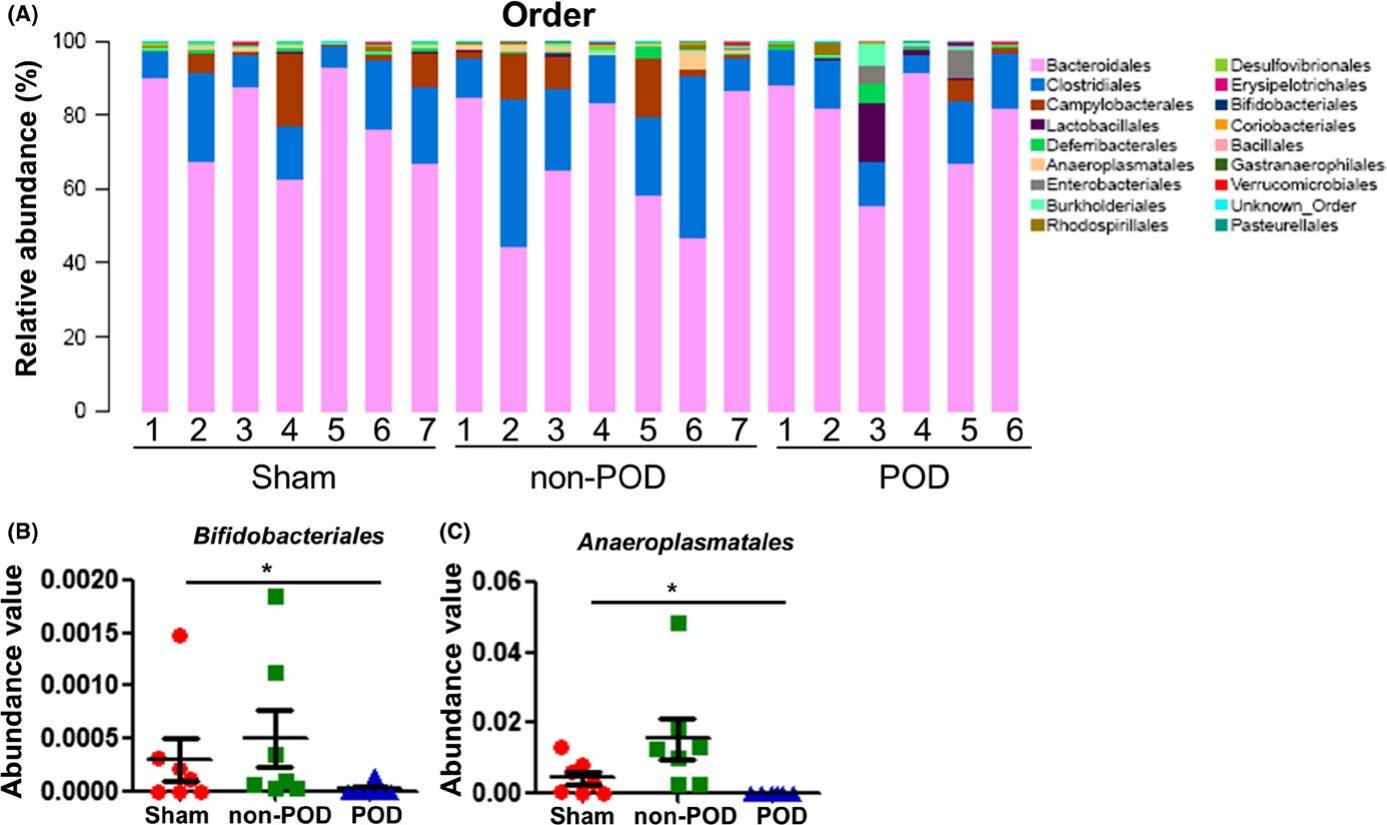
Changes in gut microbiota composition at the order level. A, Chart of the relative abundance of the differential levels of bacteria at the order level. B, Bifidobacteriales level (Fisher's exact test, *P < 0.05). C, Anaeroplasmatales level (Fisher's exact test, *P < 0.05)

The relative abundance chart (Figure 5A) of gut microbiota composition at the order level is shown. Both Bifidobacteriales and Anaeroplasmatales at the order level were significantly altered among the three groups. Interestingly, the levels of both Bifidobacteriales and Anaeroplasmatales were significantly decreased in the POD mice compared with the non‐POD mice (Figure 5B,C).

### 3.6 Alterations in gut microbiota composition at the family level

**Figure 6:**
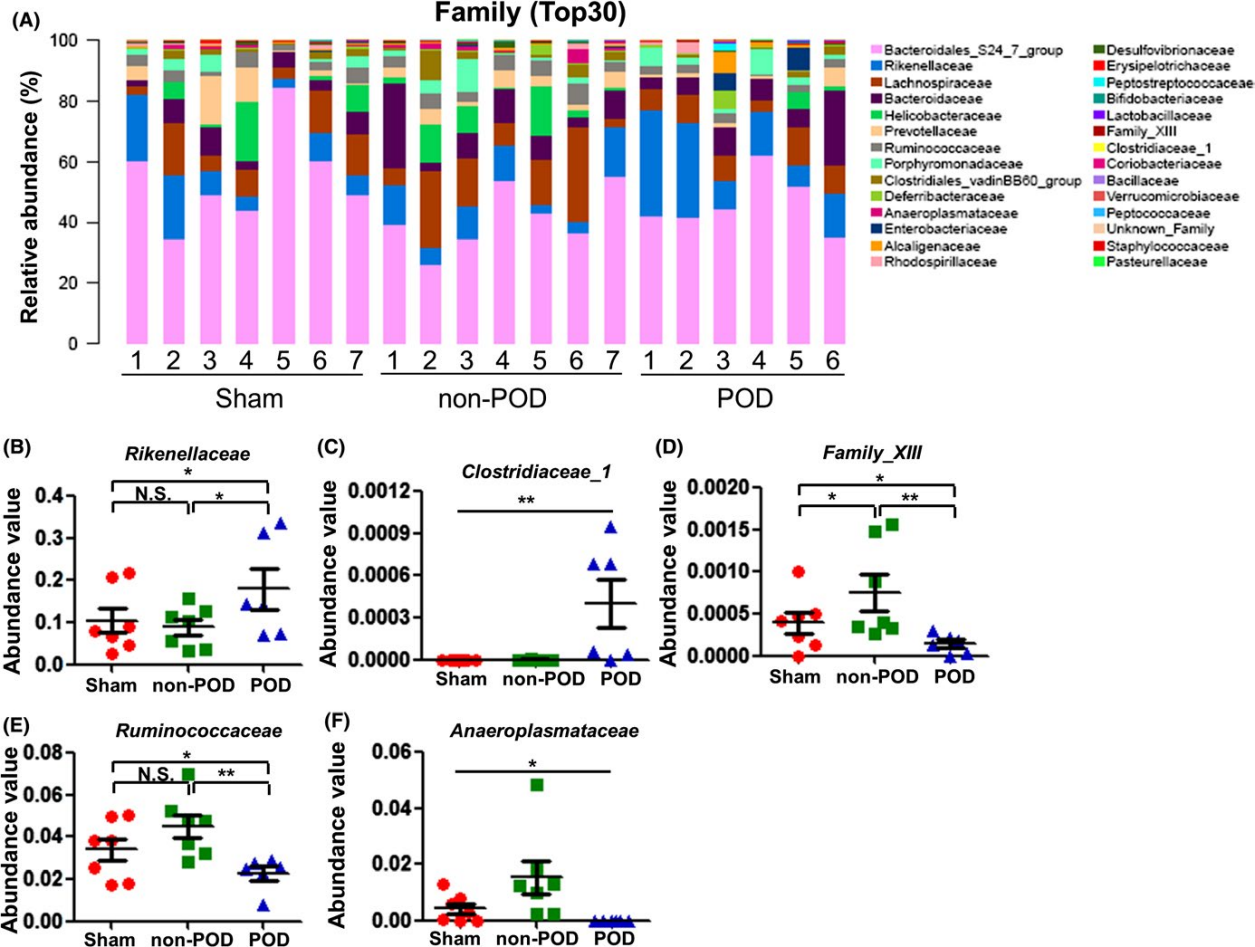
Changes in gut microbiota composition at the family level. A, Chart of the relative abundance of the differential levels of bacteria at the family level (top 30). B, Rikenellaceae level (one‐way ANOVA, *F*_2,17_ = 3.796, *P* = 0.043). C, Clostridiaceae 1 level (Fisher's exact test, *P < *0.01). D, Family XIII level (one‐way ANOVA, *F*_2,17_ = 3.796, *P* = 0.038). E, Ruminococcaceae level (one‐way ANOVA, *F*_2,17_ = 5.115, *P* = 0.018). F, Anaeroplasmataceae level (Fisher's exact test, *P < *0.01). *P < 0.05; **P < 0.01

The relative abundance chart (Figure 6A) of gut microbiota composition at the family level is shown. One‐way ANOVA demonstrated a significant change in the level of Rikenellaceae among the three groups. Further analysis suggested that the level of Rikenellaceae was significantly increased in the POD mice compared with the sham and non‐POD mice (Figure 6B). Interestingly, Clostridiaceae 1 failed to be measured in the sham and non‐POD mice but not in the POD mice (Figure 6C). Additionally, the results showed that the POD mice had significantly decreased levels of Family XIII and Ruminococcaceae compared with the non‐POD mice (Figure 6D,E). Furthermore, the level of Anaeroplasmataceae was significantly altered, and Anaeroplasmataceae was not detected in the fecal samples of the POD mice (Figure 6F).

### 3.7 Alterations in gut microbiota composition at the genus level

**Figure 7:**
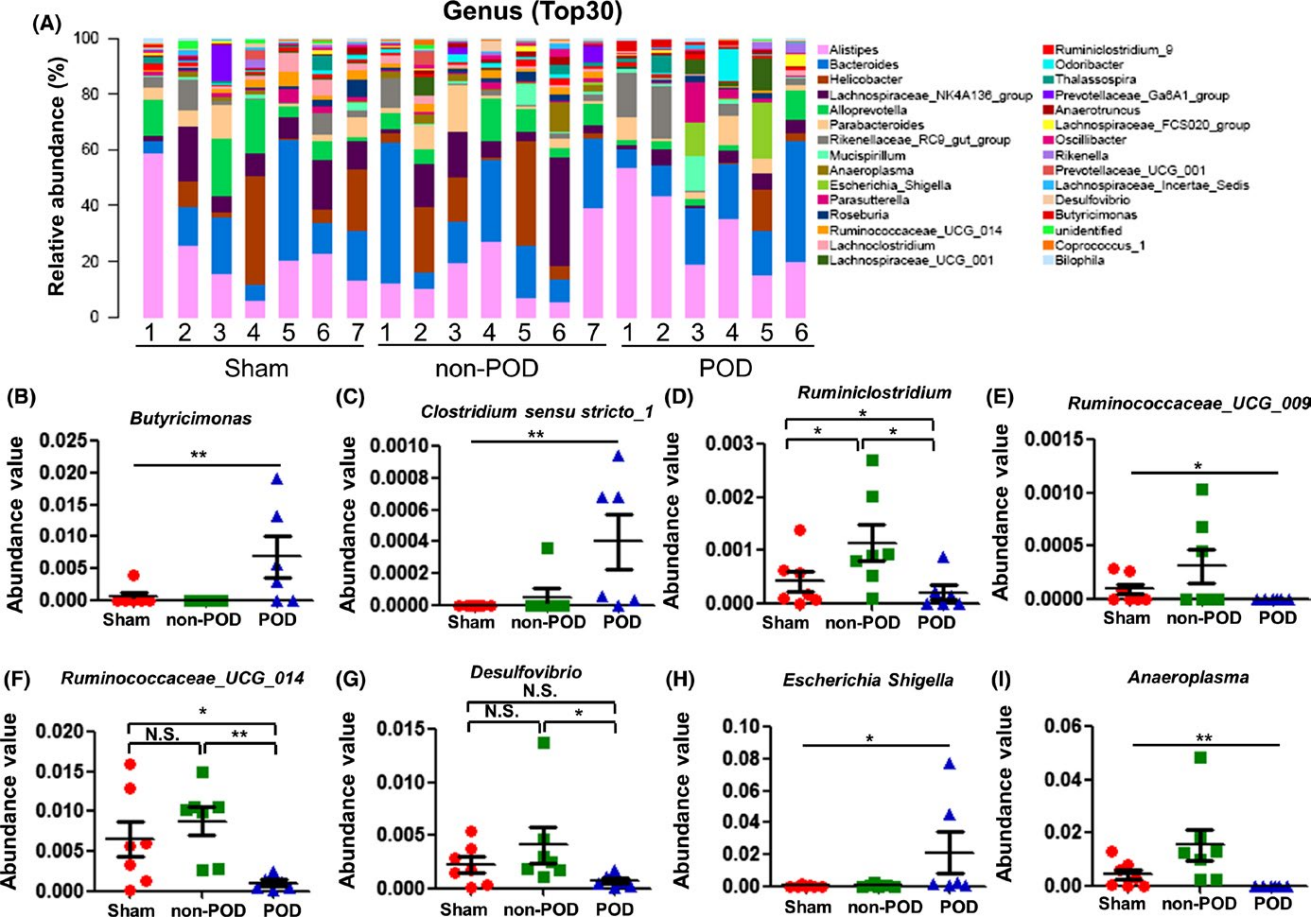
Changes in gut microbiota composition at the genus level. A, Chart of the relative abundance of the differential levels of bacteria at the genus level (top 30). B, *Butyricimonas* level (Fisher's exact test, *P < *0.01). C, *Clostridium sensu strict 1* level (Fisher's exact test, *P < *0.01). D, *Ruminiclostridium* level (one‐way ANOVA, *F*_2,17_ = 3.885, *P* = 0.041). E, *Ruminococcaceae UCG 009* level (Fisher's exact test, *P < *0.05). F, *Ruminococcaceae UCG 014* level (one‐way ANOVA, *F*_2,17_ = 5.132, *P* = 0.018). G, *Desulfovibrio* level (one‐way ANOVA, *F*_2,17_ = 3.667, *P* = 0.047). H, *Escherichia Shigella* (Fisher's exact test, *P < *0.05). I, *Anaeroplasma* level (Fisher's exact test, *P < *0.01). *P < 0.05; **P < 0.01

The relative abundance chart (Figure 7A) of gut microbiota composition at the genus level is shown. We found that the levels of *Butyricimonas*,* Clostridium sensu strict 1*,* Ruminococcaceae UCG 009*,* Escherichia Shigella*, and *Anaeroplasma* were significantly altered among the three groups (Figure 7B‐F). Furthermore, the POD mice showed significant decreases in the levels of *Ruminiclostridium*,* Ruminococcaceae UCG 014*, and *Desulfovibrio* compared with the non‐POD mice (Figure 7G‐I).

### 3.8 Alterations in gut microbiota composition at the species level

**Figure 8:**
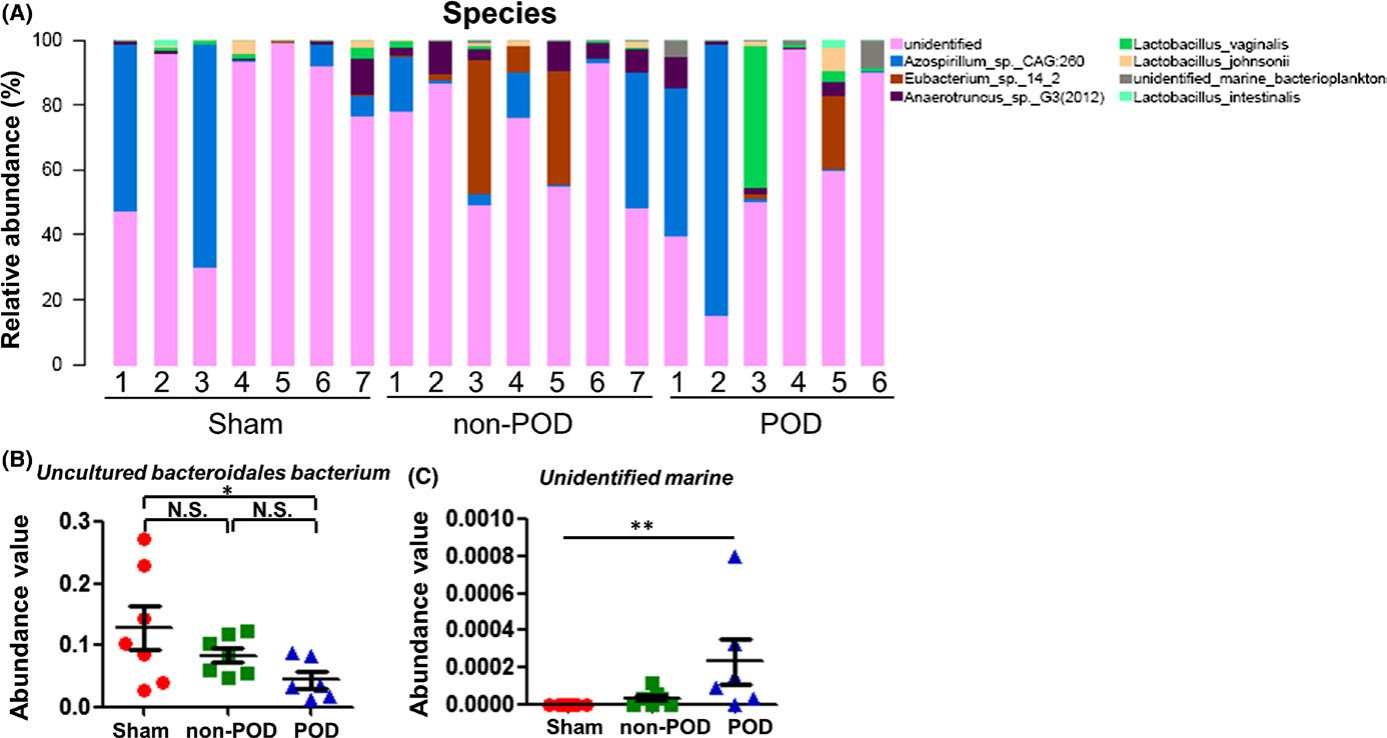
Changes in gut microbiota composition at the species level. A, Chart of the relative abundance of the differential levels of bacteria at the species level. B, *Uncultured Bacteroidales* bacterium level (one‐way ANOVA, *F*_2,17_ = 3.781, *P* = 0.044). C, *Unidentified marine* level (Fisher's exact test, *P < *0.01). *P < 0.05; **P < 0.01

The relative abundance chart (Figure 8A) of gut microbiota composition at the species level is shown. The POD mice showed significant decreases in the level of *Uncultured Bacteroidales bacterium* compared with the sham mice (Figure 8B). Additionally, the level of *Unidentified marine* was significantly altered among the three groups (Figure 8C).

### 3.9 Effects of gut microbiota transplants on behaviors in pseudo‐germ‐free mice

**Figure 9:**
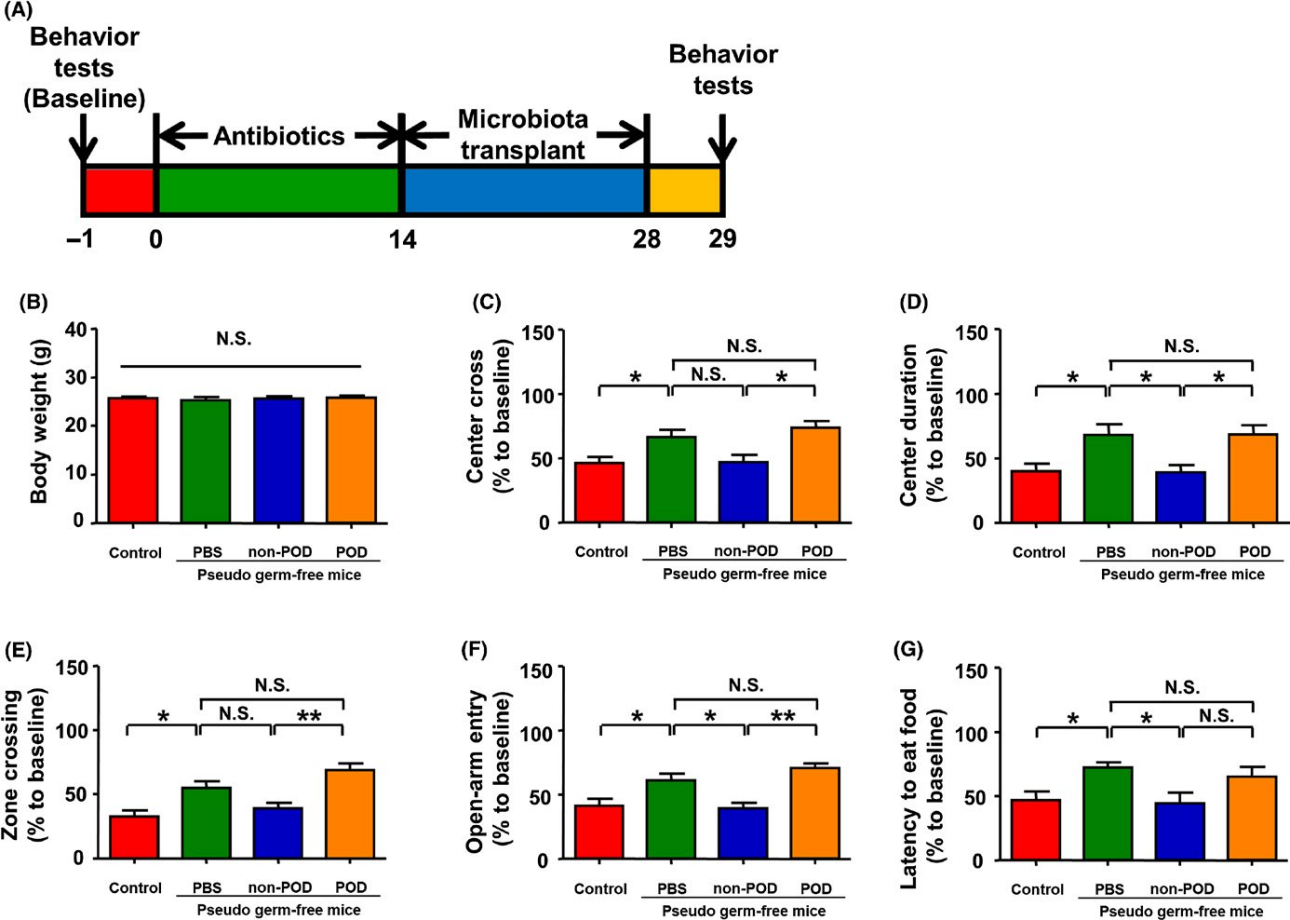
Effects of transplanting fecal bacteria from non‐POD and POD mice on the behavior of pseudo‐germ‐free mice. A, Schedule of fecal bacteria transplantation and behavior tests for the pseudo‐germ‐free mice. The pseudo‐germ‐free model was achieved by treating mice with large doses of antibiotic solution in their drinking water for 14 consecutive days. The mice were then orally treated with fecal bacteria from non‐POD or POD mice. The behavioral tests were performed on day 29. B, Body weight (one‐way ANOVA, *F*_3,41_ = 25.59, *P* < 0.001). C, Center crossing (one‐way ANOVA, *F*_3,41_ = 5.803, *P* = 0.002). D, Time spent at the center (one‐way ANOVA, *F*_3,41_ = 6.746, *P* < 0.001). E, Zone crossing (one‐way ANOVA, *F*_3,41_ = 8.143, *P* < 0.001). F, Entries into the open arms (one‐way ANOVA, *F*_3,41_ = 8.442, *P* < 0.001). G, Latency to find the food pellet (one‐way ANOVA, *F*_3,41_ = 5.059, *P* = 0.0041). ANOVA, analysis of variance; NS, not significant; PBS, phosphate‐buffered saline; POD, postoperative delirium. Data are shown as mean ± SEM (n = 11). *P < 0.05, **P < 0.01

Fourteen days after antibiotic treatment, gut microbiota from the non‐POD and POD mice was transplanted for 14 consecutive days in pseudo‐germ‐free mice (Figure 9A). On day 29, the body weight did not show a significant change among the four groups. Pseudo‐germ‐free mice showed abnormal behaviors in OFT, EPMT, and BFT. Interestingly, the pseudo‐germ‐free mice that received fecal bacteria transplants from the non‐POD mice but not from the POD mice showed improvements in behaviors (Figure 9B‐G).

## 4 DISCUSSION

In the present study, we found that α‐diversity and β‐diversity were quite different between the POD and non‐POD mice. Further analysis revealed that gut bacteria at the six levels were significantly different between the POD and non‐POD mice. These findings suggest that abnormal gut microbiota composition contributes to the underlying mechanisms of POD. To the best of our knowledge, this is the first study reporting the relationship between gut microbiota and POD, an anesthesia‐ and surgery‐related neurological complication.

Chao 1 is an index of species richness, unrelated to abundance and evenness.^26^ The Chao 1 index was decreased in the POD mice, suggesting that the number of gut microbiota in the POD mice was less. The Shannon index is related to not only species richness but also species evenness.^26^ Additionally, the Simpson index describes the probability that the number of individuals obtained from the same two consecutive samples in a bacterium community.^26^ We found that the POD mice had decreased Shannon and Simpson indices compared with the non‐POD mice, indicating that the richness and evenness of gut bacteria in the POD mice were lower. Similarly, PD whole tree demonstrated that the diversity of gut microbiota in the POD mice was relatively poor. Interestingly, PCoA and PCA are two indicators for evaluating β‐diversity.^27^ In the present study, the PCoA and PCA analysis plots of Bray‐Curtis dissimilarity among the three groups showed that the dots of the POD mice (a1‐a7) were far away from the dots of the non‐POD mice (Figure 2F), suggesting different gut microbiota composition between the POD and non‐POD mice.

Tenericutes is a phylum of gram‐negative bacteria that contains the class Mollicutes.^28^ The levels of both Tenericutes and Mollicutes were significantly increased in the non‐POD mice, whereas the levels of both were significantly decreased in the POD mice. We found that they might play an important role in the pathological and therapeutic mechanisms of POD. Gammaproteobacteria, a class of pathogenic bacteria, has detrimental potential to cause abnormal inflammatory activation.^29^ We observed the emergence of Gammaproteobacteria in the gut of the POD mice, indirectly supporting the fact that POD pathogenesis is probably related to the Gammaproteobacteria‐mediated abnormally activated inflammatory response.

Bifidobacteriales comprises species present in the gastrointestinal tract of humans and animals.^30^ We previously reported that deficits in *Bifidobacterium* are highly associated with stress susceptibility in a mouse model of chronic social defeat stress, whereas the supplementation of *Bifidobacterium* strengthens stress resilience.^31^ Additionally, Kobayashi et al^32^ demonstrated that the administration of *Bifidobacterium* to mice with Alzheimer's disease‐like phenotype reverses cognitive impairment. Consistent with these results, we found that Bifidobacteriales was significantly upregulated in the non‐POD mice, whereas the POD mice showed significant decreases in the level of Bifidobacteriales. It seems likely that the decreased level of gut Bifidobacteriales contributes to the pathogenesis of POD. Therefore, the supplementation of Bifidobacteriales prior to surgery may prevent the onset of POD.

Our previous study reported that the level of the family Ruminococcaceae was significantly altered in depression and antidepressant effects of R‐ketamine and lanicemine in mice.^33^ Interestingly, in the present study, we detected decreased levels of Ruminococcaceae and increased levels of Rikenellaceae in the fecal samples of the POD mice. Furthermore, Clostridiaceae 1 is a series of pathogenic bacteria, and our results demonstrated that the emergence of Clostridiaceae 1 promotes the onset of POD. Although there are few studies on Family XIII and Anaeroplasmataceae, their low levels may be related to the occurrence of POD. Collectively, abnormalities in the levels of Ruminococcaceae, Rikenellaceae, Clostridiaceae 1, Family XIII, and Anaeroplasmataceae might, at least partially, participate in the pathogenesis of POD.

We previously reported that an increased level of *Butyricimonas* might contribute to the antidepressant effects of R‐ketamine.^34^ Although details of the physiological actions of *Butyricimonas* are unclear, we suggest that POD is related to the increased levels of *Butyricimonas*. The levels of *Ruminiclostridium, Ruminococcaceae UCG 009*, and *Ruminococcaceae UCG 014* were significantly decreased in the gut of the POD mice compared with that of the non‐POD mice, suggesting that supplementations of these bacteria might exert preventive and therapeutic effects on POD. In addition, an increased level of *Desulfovibrio* has been detected in children with autism.^35^ Our results demonstrated that the level of *Desulfovibrio* was significantly decreased in the POD mice compared with the non‐POD mice, although the exact mechanisms are still unclear. *Escherichia Shigella* is one of the leading pathogenic causes of diarrhea, affecting an estimated 80‐165 million individuals.^36^ The POD mice were associated with increased levels of *Escherichia Shigella*, indicating that dysbiosis of gut microbiota is probably involved in the pathogenesis of POD. Further detailed studies on the role of these bacteria in the underlying mechanisms of POD are needed.

Germ‐free animals are a useful experimental model for investigating the effects of specific microbiota transplants on host physiologic, metabolic, and behavioral actions.^37^ In the present study, we used large doses of antibiotics to model pseudo‐germ‐free mice rather than absolute germ‐free mice because the latter are not likely to perform behavioral tests in non‐germ‐free environments. A previous study reported that >90% of gut microbiota would be killed by antibiotics and that the behaviors of pseudo‐germ‐free mice are similar to those of absolute germ‐free mice.^38^ Interestingly, we found that pseudo‐germ‐free mice showed abnormal behaviors. However, fecal bacteria transplants from the non‐POD mice, but not from the POD mice, improved the abnormal behaviors in the pseudo‐germ‐free mice. To the best of our knowledge, this is the first study showing the effects of non‐POD and POD fecal transplants on behaviors in pseudo‐germ‐free mice. These findings suggest that gut microbiota has physiological potential to affect behavioral performance.

## 5 CONCLUSION

Abnormal gut microbiota composition may contribute to the pathogenesis of POD. Because the diagnosis of POD is currently dependent on clinical symptoms, detecting gut microbiota may provide an accurate alternative to diagnosis. In addition, we suggest that supplementation with physiologically beneficial bacteria and/or the accurate removal of pathogenic bacteria will provide a novel preventive and therapeutic approach for POD treatment. Future studies, particularly clinical trials, are required to explore the potential pathological and therapeutic roles of gut microbiota in POD.

## CONFLICT OF INTEREST

The authors declare no conflict of interest.
