# Supplementary material for: Abnormal composition of gut microbiota contributes to delirium‐like behaviors after abdominal surgery in mice
Source: CNS Neurosci Ther. 2019 Jan 24;25(6):685–96. doi: 10.1111/cns.13103 (PMC6515708; doi:10.1111/cns.13103)
Supplement: Supplementary file 1 [file CNS-25-685-s001.docx]

| **sample ID** | **clean tags** | **valid tags** | **valid%** | **Min Length** | **Mean Length** | **Max Length** | **OTU counts** | **subseq indeepth30860 OUT counts** |
| --- | --- | --- | --- | --- | --- | --- | --- | --- |
| **control 1** | **41373** | **35611** | **86.07%** | **414** | **434.48** | **442** | **141** | **138** |
| **control 2** | **39233** | **33482** | **85.34%** | **412** | **429.87** | **442** | **180** | **178** |
| **control 3** | **38341** | **32443** | **84.61%** | **412** | **433.83** | **464** | **179** | **177** |
| **control 4** | **38257** | **33852** | **88.48%** | **409** | **429.25** | **442** | **242** | **241** |
| **control 5** | **40078** | **33810** | **84.36%** | **414** | **434.46** | **470** | **171** | **170** |
| **control 6** | **41954** | **35634** | **84.93%** | **413** | **431.63** | **442** | **204** | **204** |
| **control 7** | **40873** | **33892** | **82.92%** | **412** | **429.99** | **442** | **233** | **230** |
| **non-POD 1** | **41770** | **34624** | **82.89%** | **413** | **433.25** | **442** | **211** | **209** |
| **non-POD 2** | **41485** | **33761** | **81.38%** | **64** | **425.86** | **442** | **211** | **208** |
| **non-POD 3** | **39818** | **33618** | **84.42%** | **365** | **429.80** | **472** | **223** | **223** |
| **non-POD 4** | **38846** | **32854** | **84.57%** | **410** | **433.28** | **442** | **197** | **197** |
| **non-POD 5** | **41625** | **34384** | **82.60%** | **329** | **428.83** | **467** | **212** | **211** |
| **non-POD 6** | **40777** | **33835** | **82.97%** | **412** | **426.36** | **442** | **239** | **236** |
| **non-POD 7** | **39399** | **33926** | **86.10%** | **411** | **433.89** | **444** | **184** | **182** |
| **POD 1** | **38636** | **30861** | **79.87%** | **348** | **434.02** | **442** | **152** | **152** |
| **POD 2** | **38198** | **32245** | **84.41%** | **411** | **432.80** | **442** | **169** | **169** |
| **POD 3** | **41777** | **36784** | **88.04%** | **411** | **435.08** | **443** | **152** | **147** |
| **POD 4** | **41110** | **33883** | **82.42%** | **413** | **434.98** | **442** | **142** | **139** |
| **POD 5** | **38419** | **32919** | **85.68%** | **410** | **431.89** | **468** | **220** | **218** |
| **POD 6** | **39818** | **33655** | **84.52%** | **411** | **432.78** | **468** | **154** | **150** |
